# Lesion-Specific Clinical Implications of Oral Hesitation After Stroke: A Comparative Study of Frontal Versus Parietal Lobe Lesions

**DOI:** 10.3390/medicina62050918

**Published:** 2026-05-09

**Authors:** Yeo Joon Yun, Seong Ho Jang, Jae Hyeon Park, Seung Yoon Choi, Ji Woo Lee, Seung Hoon Han

**Affiliations:** 1 Department of Rehabilitation Medicine, Hanyang University Guri Hospital, Guri-si 11923, Republic of Korea; yunyujun@naver.com (Y.J.Y.);; 2 Department of Rehabilitation Medicine, Hanyang University Medical Center, Seoul 04763, Republic of Korea

**Keywords:** dysphagia, stroke, oral hesitation, frontal lobe, parietal lobe, penetration-aspiration scale, chewing, rehabilitation

## Abstract

*Background and Objectives*: Whether post-stroke oral hesitation carries different clinical implications by lesion location is unclear. We compared oral hesitation and its relationship with chewing, cognition, and aspiration risk between frontal and parietal lobe stroke. *Materials and Methods*: We retrospectively analyzed 242 patients (35 frontal, 207 parietal) from 946 consecutive stroke admissions (2016–2020) with isolated lesions and videofluoroscopic swallowing study within one month. Oral hesitation, chewing, Clinical Dysphagia Scale (CDS), and Mini-Mental State Examination (MMSE) were recorded. Penetration-Aspiration Scale (PAS) scores were categorized as Normal (1), Penetration (2–5), or Aspiration (6–8). Multivariable logistic regression adjusting for age, sex, stroke type, and lesion side was performed. Firth’s penalized estimation was used for models with quasi-separation. *Results*: Groups were demographically comparable in age (68.1 ± 15.0 vs. 71.7 ± 12.2 years; *p* = 0.206) and female sex (48.6% vs. 42.0%; *p* = 0.590). Oral hesitation was significantly more prevalent in the frontal group (liquid: 80.0% vs. 23.2%, *p* < 0.001; semisolid: 68.6% vs. 26.6%, *p* < 0.001). Frontal patients scored worse on six of seven CDS subcomponents (*p* < 0.01), yet chewing was uncorrelated with oral hesitation or residue (*p* > 0.3), unchanged after MMSE adjustment. In parietal patients, chewing correlated with all outcomes (ρ = 0.19–0.30, *p* < 0.01). In parietal stroke, oral hesitation was linked with liquid aspiration (64.3% vs. 35.7%; OR = 3.25, *p* = 0.001) and semisolid airway invasion (OR = 2.70, *p* = 0.005); these associations remained significant after multivariable adjustment and FDR correction. No such association was detected in the frontal group, although this finding is limited by the smaller sample size. *Conclusions*: Oral hesitation may carry different clinical implications by lesion site. In parietal stroke, it was associated with chewing impairment and higher aspiration risk, suggesting a possible sensorimotor contribution. Frontal group findings were underpowered and should be considered exploratory. Lesion-specific interpretation warrants larger-cohort confirmation.

## 1. Introduction

Post-stroke dysphagia is a common and clinically significant complication, occurring in 33 to 77 percent of stroke patients and contributing substantially to aspiration pneumonia, malnutrition, and reduced quality of life [1,2]. Inadequate nutrition during stroke recovery has also been shown to worsen functional outcomes [3]. Among the various manifestations of post-stroke dysphagia, oral hesitation, defined as delayed initiation or impaired coordination of bolus propulsion in the oral phase, has attracted increasing attention as a clinically identifiable and measurable phenomenon [4,5].

The neural substrates underlying oral hesitation are beginning to be clarified. Swallowing is controlled by a distributed cortical network that includes primary sensorimotor, insular, frontal opercular, and posterior parietal regions [6,7]. Lesions at different nodes within this network produce distinct dysphagia phenotypes [8,9]. Our previous study in patients with isolated frontal lobe stroke demonstrated that oral hesitation was significantly associated with cognitive impairment, particularly during liquid swallowing, suggesting that executive or attentional factors may contribute to this deficit in frontal lesions [10]. In parallel, parietal lobe lesions have been implicated in disrupting oral sensorimotor integration, given the parietal lobe’s well-established role in tactile processing, bolus shape recognition, and motor planning [11,12]. These distinct anatomical roles raise the possibility that oral hesitation, although phenotypically similar across lesions, may have different underlying characteristics [13].

Despite these converging lines of evidence, a direct comparison of oral hesitation patterns between frontal and parietal stroke has not been performed. Specifically, it remains unclear whether chewing ability, a core oral motor function that integrates sensory and motor control, is associated with oral hesitation in the same manner across lesion sites. Equally importantly, the clinical significance of oral hesitation has not been systematically compared between groups: whether oral hesitation predicts aspiration risk, as measured by the Penetration-Aspiration Scale (PAS) [14,15], may differ by lesion location.

Clarifying these questions has direct clinical relevance. If oral hesitation shows different patterns in frontal versus parietal stroke, dysphagia assessment and aspiration precautions could benefit from lesion-specific interpretation [16]. In particular, if oral hesitation more reliably predicts aspiration risk in one group, the same symptom may warrant different clinical responses depending on lesion location.

The present study therefore aimed to compare frontal and parietal lobe stroke cohorts with respect to four questions. First, whether the frequency of oral hesitation differs between frontal and parietal stroke. Second, whether chewing ability is associated with oral hesitation and pharyngeal residue in the same manner across groups. Third, whether the relationship between cognition and oral hesitation is modified by lesion site. Fourth, whether oral hesitation is associated with aspiration risk, as indexed by PAS-based categorization, to a similar degree in both groups.

## 2. Materials and Methods

### 2.1. Study Design and Participants

This retrospective observational study was conducted using medical records and videofluoroscopic swallowing study (VFSS) data from patients hospitalized at Hanyang University Seoul Hospital and Hanyang University Guri Hospital between January 2016 and December 2020. A total of 946 consecutive stroke admissions were screened for eligibility. The study was approved by the Institutional Review Board of Hanyang University Guri Hospital (IRB No. 2025-09-036; approved on 25 September 2025), and the requirement for informed consent was waived owing to the retrospective nature of the study and the use of de-identified data. All procedures conformed to the principles of the Declaration of Helsinki.

Patients were included if they had (1) a diagnosis of stroke restricted exclusively to the frontal lobe or the parietal lobe, confirmed on magnetic resonance imaging (diffusion-weighted and fluid-attenuated inversion recovery sequences) or computed tomography; and (2) a VFSS performed within one month of stroke onset. Patients were excluded if they had pre-existing swallowing disorders, coexisting neurological diseases other than stroke, severe cognitive impairment precluding VFSS cooperation, or missing VFSS records.

Demographic characteristics of the frontal cohort have been described in part in our earlier work on cognition and oral hesitation [10]; however, the chewing, CDS subcomponent, PAS, and comparative analyses reported in the present study were not examined in that earlier work.

### 2.2. Videofluoroscopic Swallowing Study

VFSS was performed by trained rehabilitation physicians following a standardized institutional protocol. Recordings were obtained in the lateral plane using fluoroscopy (Shimadzu Corporation, Kyoto, Japan) at 15 to 30 frames per second. Patients sequentially received five bolus consistencies: thin liquid (3 mL of diluted barium), thick liquid (yogurt consistency), semisolid (soft pudding, 5 mL of barium), rice porridge, and solid food (a spoonful of rice). Because few patients safely progressed beyond the semisolid trial, primary analyses focused on the thin liquid (Stage 1) and semisolid (Stage 3) trials. Tests were terminated if patients exhibited significant difficulty or a high risk of aspiration at any stage.

### 2.3. Oral Hesitation Assessment

Oral hesitation was defined as delayed initiation or impaired coordination of oral bolus propulsion and was assessed separately for liquid and semisolid consistencies, following the operational definitions used in our prior work on frontal lobe stroke [10] and consistent with published normative timing values for oropharyngeal swallowing [17]. All VFSS recordings were reviewed by two trained rehabilitation physicians; assessors were not blinded to lesion site, as clinical information was available during routine VFSS interpretation. For liquid swallowing, oral hesitation was defined as an oral transit time exceeding 1.5 seconds (normal reference: 0.35 to 1.5 seconds). For semisolid swallowing, oral hesitation was defined as either (a) a delay of more than 1.5 seconds before initiation of mastication, or (b) a total oral phase duration exceeding 9 seconds before pharyngeal transfer. Presence of oral hesitation was coded as a binary variable (0 = absent, 1 = present) for each consistency.

### 2.4. Clinical Dysphagia Scale and Pharyngeal Residue

Oral motor function was evaluated using the Clinical Dysphagia Scale (CDS), which includes seven subcomponents rated on ordinal scales: lip sealing, chewing and mastication, tongue protrusion, laryngeal elevation, reflex coughing, residue in mouth, and velar elevation. CDS scoring was performed by trained rehabilitation physicians who had completed standardized training on the instrument; however, formal inter-observer reliability data were not collected in this study. The CDS is a validated tool with established reliability and has been widely used in similar retrospective VFSS studies. Pharyngeal residue in the vallecular and pyriform sinuses was graded on a four-point scale (0 = none, 1 = filling less than 10 percent of the recess, 2 = 10 to 50 percent, 3 = greater than 50 percent).

### 2.5. Penetration-Aspiration Scale and Airway Invasion Classification

Aspiration risk was quantified using the 8-point Penetration-Aspiration Scale (PAS) originally developed by Rosenbek and colleagues [14], applied separately to thin liquid and semisolid trials. Higher PAS scores indicate greater airway compromise. Following the original definitions [14,15], PAS scores were categorized into three clinically meaningful groups: (a) Normal (PAS = 1; no material entering the airway); (b) Penetration (PAS = 2 to 5; material entering the larynx but remaining at or above the level of the vocal folds); and (c) Aspiration (PAS = 6 to 8; material passing below the level of the vocal folds). The term “airway invasion” was used to denote any PAS score of 2 or higher (i.e., penetration or aspiration combined), whereas “aspiration” specifically referred to PAS scores of 6 to 8. This categorization was used to avoid conflating penetration and aspiration, which have distinct anatomical and clinical implications.

### 2.6. Cognitive Assessment

Cognitive function was assessed using the Korean version of the Mini-Mental State Examination (MMSE) [18]. In secondary analyses, an MMSE score below 24 was used to classify patients as cognitively impaired, consistent with the threshold used in our prior work [10].

### 2.7. Statistical Analysis

Continuous variables are presented as mean ± standard deviation and were compared between groups using the Mann–Whitney U test. Categorical variables are presented as frequencies and percentages and were compared using the chi-square test or Fisher’s exact test as appropriate; for categorical variables with low expected cell counts (<5), Fisher’s exact test was implemented using Monte Carlo permutation with 10,000 replications. Associations between chewing ability, oral hesitation, and pharyngeal residue were assessed with Spearman’s rank correlation. To examine whether associations were independent of cognitive status, partial Spearman correlations adjusted for MMSE score were computed within each group using rank-based residualization. To test whether the effect of lesion group on oral hesitation was modified by cognition, logistic regression models including a Group × MMSE interaction term were fitted for both liquid and semisolid oral hesitation; Firth’s penalized maximum likelihood estimation was used to address quasi-separation resulting from the small frontal sample. The association between oral hesitation and PAS-defined airway invasion (Aspiration [PAS 6 to 8] or any airway invasion [PAS ≥ 2]) was assessed using Fisher’s exact test with odds ratios (ORs) and 95% confidence intervals (CIs) reported within each group. To evaluate the independent effect of oral hesitation on aspiration risk after adjusting for potential confounders, multivariable logistic regression models were fitted with age, sex, stroke type (hemorrhagic versus ischemic), and lesion side as covariates; a Group × oral hesitation interaction term was included to test whether the OH–aspiration association differed by lesion group. To address the imbalance in stroke type distribution between the frontal and parietal cohorts, sensitivity analyses were performed within the frontal cohort comparing oral hesitation prevalence and OH–airway invasion associations between hemorrhagic and ischemic subgroups (Supplementary Tables S1 and S2). To control for the risk of type I error due to multiple comparisons, Benjamini–Hochberg false discovery rate (FDR) correction was applied within each family of related tests. Statistical significance was set at *p* < 0.05 (two-tailed). Analyses were performed using Python 3.11 (Python Software Foundation, Wilmington, DE, USA) with SciPy version 1.11.4 and statsmodels version 0.14.1.

## 3. Results

### 3.1. Baseline Characteristics

A total of 242 patients were included in the final analysis. The frontal (*n* = 35) and parietal (*n* = 207) groups did not differ significantly with respect to age (68.1 ± 15.0 vs. 71.7 ± 12.2 years; *p* = 0.206), female sex (48.6 percent vs. 42.0 percent; *p* = 0.590), MMSE score (15.3 ± 10.5 vs. 15.6 ± 9.3; *p* = 0.942), or total CDS score (26.8 ± 19.6 vs. 22.4 ± 16.8; *p* = 0.334) (Table 1). However, the two groups differed significantly in lesion side distribution (*p* < 0.001), with a higher proportion of bilateral lesions in the frontal cohort (14.3 percent vs. 1.0 percent), and in stroke type (*p* < 0.001), with hemorrhagic lesions predominating in the frontal cohort (68.6 percent vs. 7.7 percent) and ischemic lesions predominating in the parietal cohort. These differences reflect the natural epidemiology of isolated lesion subtypes and are addressed in Section 3.2 and Section 4. Oral hesitation was significantly more prevalent in the frontal group for both liquid (80.0 percent vs. 23.2 percent; *p* < 0.001) and semisolid consistencies (68.6 percent vs. 26.6 percent; *p* < 0.001).

### 3.2. Sensitivity Analysis by Stroke Type Within the Frontal Cohort

Because the frontal and parietal cohorts differed significantly in stroke type distribution (Table 1), we first performed sensitivity analyses within the frontal cohort to evaluate whether any lesion-specific patterns observed in the subsequent analyses could be attributable to the higher proportion of hemorrhagic lesions in the frontal group. Oral hesitation prevalence did not differ significantly between the frontal-hemorrhage (*n* = 24) and frontal-infarction (*n* = 11) subgroups for either liquid (79.2 percent vs. 81.8 percent; *p* = 1.000) or semisolid swallowing (62.5 percent vs. 81.8 percent; *p* = 0.435) (Supplementary Table S1). Within the frontal-hemorrhage subgroup, oral hesitation was not significantly associated with liquid aspiration (44.4 percent in OH+ vs. 60.0 percent in OH−; OR = 0.53, *p* = 0.640) (Supplementary Table S2), and the descriptive pattern in the frontal-infarction subgroup also did not show a clear OH–aspiration relationship, although this subgroup was too small for stable statistical estimation (OH− subgroup *n* = 1). These findings suggest that the imbalance in stroke type distribution is unlikely to fully explain the patterns observed in the main analyses, although this analysis was limited by small subgroup size.

### 3.3. CDS Subcomponent Profile

Comparison of the seven CDS subcomponents revealed a pronounced pattern: frontal stroke patients showed significantly worse scores on six of the seven subcomponents, including lip sealing (*p* < 0.001), chewing and mastication (*p* = 0.001), tongue protrusion (*p* = 0.001), laryngeal elevation (*p* = 0.002), residue in mouth (*p* < 0.001), and velar elevation (*p* < 0.001) (Table 2). Only reflex coughing did not differ between groups (*p* = 0.789). These findings indicate substantially more severe oral motor involvement in the frontal cohort, an important baseline for interpreting subsequent analyses.

### 3.4. Chewing Ability and Swallowing Outcomes: A Group-Dependent Pattern

Despite significantly worse chewing scores at the group level, chewing ability in the frontal group showed no significant correlation with any swallowing outcome: oral hesitation during liquid (ρ = −0.023, *p* = 0.898), oral hesitation during semisolid (ρ = 0.152, *p* = 0.389), vallecular residue (ρ = −0.118, *p* = 0.512), or pyriform residue (ρ = 0.141, *p* = 0.435) (Table 3). In contrast, chewing ability in the parietal group was significantly associated with every swallowing outcome: liquid oral hesitation (ρ = 0.185, *p* = 0.008), semisolid oral hesitation (ρ = 0.251, *p* < 0.001), vallecular residue (ρ = 0.301, *p* < 0.001), and pyriform residue (ρ = 0.250, *p* < 0.001). To examine whether the absence of chewing-outcome correlations in frontal stroke could be attributed to cognitive confounding, we additionally computed MMSE-adjusted partial Spearman correlations within the frontal group (Table 3, right column). All four associations remained non-significant after MMSE adjustment, suggesting that the observed pattern in frontal stroke is not primarily explained by shared cognitive variance. However, this null finding should be interpreted cautiously given the smaller frontal sample.

### 3.5. Lesion Group and the Cognitive Effect on Oral Hesitation

To test whether the effect of cognition on oral hesitation differed between groups, logistic regression models with a Group × MMSE interaction term were fitted using Firth’s penalized maximum likelihood estimation to address quasi-separation in the small frontal sample (Table 4). For liquid oral hesitation, the lesion group effect was significant (*p* = 0.001), and the Group × MMSE interaction did not reach significance (*p* = 0.068). The negative interaction coefficient suggests that higher MMSE scores were associated with a larger reduction in oral hesitation risk in the frontal group than in the parietal group, although this effect was not statistically significant. The Group main effect OR was 122.2 (95% CI 6.7–2243.9) using Firth correction, compared with 265.3 in the standard model; the wide confidence interval reflects the strong group separation in oral hesitation prevalence and should be interpreted as descriptive evidence of a large group difference rather than a precise effect estimate. For semisolid oral hesitation, the group effect was also significant (*p* = 0.002), but the Group × MMSE interaction did not reach significance (*p* = 0.209).

### 3.6. Penetration-Aspiration Profile and Its Relationship with Oral Hesitation

The distribution of PAS categories by group is presented in Table 5. Overall aspiration rates (PAS 6 to 8) did not differ significantly between groups for either liquid (frontal 57.6 percent vs. parietal 41.7 percent; *p* = 0.129) or semisolid trials (13.0 percent vs. 7.5 percent; *p* = 0.408). The proportion of patients exhibiting penetration (PAS 2 to 5) during liquid swallowing was significantly lower in the frontal group (6.1 percent vs. 26.6 percent; *p* = 0.008), suggesting a different distribution of airway invasion severity between groups.

**Figure 1:**
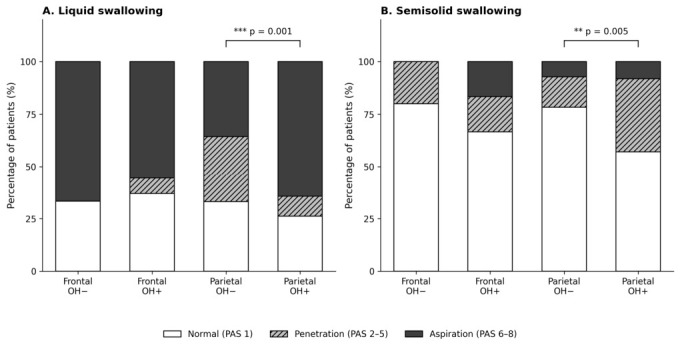
Distribution of Penetration-Aspiration Scale (PAS) categories in frontal and parietal lobe stroke patients, stratified by the presence of oral hesitation (OH), for (**A**) liquid and (**B**) semisolid swallowing. PAS scores were categorized as Normal (PAS 1, white), Penetration (PAS 2–5, hatched), and Aspiration (PAS 6–8, dark gray). Significant differences between OH+ and OH− in the parietal group are indicated. OH, oral hesitation; PAS, Penetration-Aspiration Scale. ** *p* < 0.01, *** *p* < 0.005, Fisher’s exact test.

The association between oral hesitation and PAS-defined airway invasion differed between groups (Table 6, Figure 1). In the parietal cohort, oral hesitation during liquid swallowing was significantly associated with liquid aspiration: 64.3 percent of parietal patients with oral hesitation aspirated, compared with 35.7 percent of those without oral hesitation (OR = 3.25, 95% CI 1.60–6.61, *p* = 0.001). Similarly, oral hesitation during semisolid swallowing was associated with any airway invasion (penetration or aspiration) in the parietal group (42.9 percent vs. 21.7 percent; OR = 2.70, 95% CI 1.36–5.36, *p* = 0.005). In the frontal group, we did not detect a significant association between oral hesitation and airway invasion for either consistency, with confidence intervals that were wide and crossed unity. Given the smaller frontal sample (*n* = 35) and the resulting limited statistical power, these null findings should be interpreted with caution rather than as evidence of absent association. In multivariable logistic regression adjusting for age, sex, stroke type, and lesion side, the association between oral hesitation and liquid aspiration remained significant in the overall cohort (adjusted OR = 3.35, 95% CI 1.62–6.91, *p* = 0.001). The Group × OH interaction was not significant (*p* = 0.121), suggesting that the overall OH–aspiration association was not significantly modified by lesion group after covariate adjustment, although the direction of the interaction was consistent with a stronger effect in the parietal group. For semisolid any invasion, the adjusted OH effect also remained significant (adjusted OR = 2.71, 95% CI 1.36–5.40, *p* = 0.005). All key associations survived Benjamini–Hochberg FDR correction.

## 4. Discussion

This study compared oral hesitation patterns and their relationship with chewing ability, cognition, and aspiration risk in patients with isolated frontal or parietal lobe stroke. Five principal observations emerged, corresponding to the four study aims. First, oral hesitation was significantly more prevalent in the frontal group than in the parietal group for both liquid (80.0% vs. 23.2%) and semisolid (68.6% vs. 26.6%) consistencies, establishing that the frequency of oral hesitation differs substantially by lesion site. Second, despite significantly worse oral motor scores across most CDS subcomponents in the frontal group, chewing ability was not detectably correlated with oral hesitation or pharyngeal residue in frontal stroke, whereas it was consistently associated with both in parietal stroke. Third, this pattern in frontal stroke persisted after adjustment for MMSE, and logistic regression suggested a group-dependent effect of cognition on oral hesitation. Fourth, the distribution of PAS categories differed between groups, with parietal patients showing a higher proportion of liquid penetration (PAS 2 to 5), while overall aspiration rates (PAS 6 to 8) did not differ. Fifth, and of direct clinical relevance, oral hesitation in parietal stroke was associated with a markedly elevated risk of liquid aspiration (OR = 3.25, *p* = 0.001) and of any semisolid airway invasion (OR = 2.70, *p* = 0.005), and these associations remained significant after multivariable adjustment for age, sex, stroke type, and lesion side; no such association was detected in frontal stroke, although this null finding is limited by the smaller sample size. Together, these findings suggest lesion-specific clinical implications of oral hesitation, although the smaller frontal sample limits any mechanistic interpretation and these observations should be considered preliminary.

The lack of a detectable chewing-outcome link in frontal stroke, despite marked oral motor impairment at the group level, is noteworthy. It is important to note that while total CDS scores did not differ between groups (Table 1), the individual subcomponents showed significantly worse performance in the frontal group on six of seven items (Table 2). This apparent discrepancy reflects the fact that the total CDS score aggregates heterogeneous subcomponents with different clinical weights, and group-level differences in specific oral motor functions can be masked by compensatory patterns in other subcomponents or by floor/ceiling effects. The subcomponent-level analysis is therefore more informative for understanding the specific patterns of oral motor dysfunction. One possible interpretation is that oral hesitation in frontal stroke may be more closely related to disruption of volitional initiation and executive control than to peripheral motor incompetence [4,19]. Frontal lobe lesions in stroke have been associated with delayed oral transit and impaired swallowing initiation [5,20]. Similar dissociations between oral motor integrity and swallowing initiation have been described in Alzheimer’s disease and vascular dementia [21,22]. Our previous work also demonstrated that oral hesitation in frontal stroke is closely linked to MMSE-indexed cognitive function [10]. The present analysis extends this by showing that, after adjustment for cognition, chewing still does not explain oral hesitation in frontal patients, and that oral hesitation in the frontal group did not translate into elevated aspiration risk on VFSS. If confirmed in larger studies, these observations would be compatible with a cognitive-executive interpretation, in which oral hesitation may reflect impaired swallowing initiation rather than a mechanical deficit that propagates into the pharyngeal phase, although this remains speculative. However, we caution against strong mechanistic conclusions from these null findings; with 35 frontal patients, statistical power to detect moderate correlations is limited, and larger studies will be needed to confirm whether chewing and aspiration risk are truly dissociated from oral hesitation in frontal stroke.

The decision to focus the primary correlation analysis on chewing ability, rather than on all seven CDS subcomponents, warrants explanation. Chewing and mastication was selected as the primary predictor because it is the most functionally relevant oral motor subcomponent for bolus preparation and oral phase initiation, and because it has the most direct physiological connection to oral hesitation and pharyngeal transfer. While other subcomponents (e.g., lip sealing, tongue protrusion, velar elevation) also differed significantly between groups (Table 2), they are less directly involved in the oral-to-pharyngeal transition that defines oral hesitation. A comprehensive analysis of all subcomponents would introduce substantial multiplicity concerns and would be better suited to an exploratory subcomponent-level study. Nonetheless, we acknowledge that the relationship between other CDS subcomponents and swallowing outcomes deserves further investigation. In contrast, the consistent coupling between chewing, oral hesitation, pharyngeal residue, and airway invasion in parietal stroke is compatible with a sensorimotor integration account. The parietal cortex, particularly the inferior parietal lobule and adjacent somatosensory areas, plays an important role in integrating tactile, proprioceptive, and spatial information about the bolus and in generating feedforward signals for coordinated oral motor action [7,11]. Parietal-mediated sensorimotor dysphagia has been documented in other conditions, including Huntington disease, where parieto-thalamo-cerebellar atrophy predicts compromised swallowing safety [12]. Functional connectivity studies have also shown that parietal-temporal network dysfunction correlates with the severity of post-stroke dysphagia [13]. In our cohort, the parallel associations of chewing with oral hesitation and pharyngeal residue, together with the more than threefold increase in liquid aspiration risk among parietal patients with oral hesitation, are compatible with a sensorimotor interpretation in which deficits in parietal stroke may affect both the oral and pharyngeal phases and may be associated with airway compromise [23], although this interpretation should be considered tentative given the observational design.

The clinical implication of the PAS analysis warrants particular attention. We deliberately categorized PAS scores using the original framework [14,15] into Normal (PAS 1), Penetration (PAS 2 to 5), and Aspiration (PAS 6 to 8), rather than dichotomizing at a single threshold such as PAS of 3 or more, because penetration and aspiration reflect anatomically and clinically distinct airway events. This categorization revealed a notable association in parietal stroke: oral hesitation during liquid swallowing was associated with a more than threefold increase in the odds of aspiration, and oral hesitation during semisolid swallowing was associated with a higher rate of any airway invasion. The absolute aspiration rate in parietal patients with liquid oral hesitation reached 64.3 percent, compared with 35.7 percent in those without oral hesitation, a difference likely to be clinically relevant at the bedside. We did not detect such associations in the frontal group, though the limited sample size precludes firm conclusions about the absence of association.

If replicated in larger cohorts, these findings would support a role for oral hesitation as a bedside marker of aspiration risk specifically in parietal stroke. Current post-stroke dysphagia management guidelines recommend individualized assessment and tailored intervention strategies [16], and lesion-specific consideration of oral hesitation may contribute to this personalized approach. Diet texture modification and aspiration precautions may therefore be particularly relevant when oral hesitation is observed in parietal stroke [17,23]. We also observed a distinct overall PAS distribution between the two groups, with a lower proportion of isolated liquid penetration in frontal patients, which may deserve further exploration as it could reflect different airway invasion dynamics between lesion types.

Several limitations should be acknowledged. First, the frontal cohort (*n* = 35) was substantially smaller than the parietal cohort (*n* = 207), reflecting the natural epidemiology of isolated lesion subtypes but meaningfully limiting statistical power in the frontal group. Accordingly, the absence of significant associations in the frontal group should be interpreted as absence of evidence rather than evidence of absence, and all findings related to the frontal group should be considered underpowered and exploratory. Second, the stroke-type and lesion-side distributions differed significantly between groups (both *p* < 0.001). To partially address this concern, we performed multivariable logistic regression adjusting for age, sex, stroke type, and lesion side, and the key findings remained significant. We also performed sensitivity analyses within the frontal cohort (Supplementary Tables S1 and S2), which showed consistent patterns across hemorrhagic and ischemic subgroups. Nevertheless, residual confounding cannot be excluded, and our findings should be considered hypothesis-generating. Third, VFSS assessors were not blinded to lesion site, which could introduce assessment bias; however, the VFSS protocol was standardized and the primary outcomes (PAS scores, oral transit times) are based on objective measurements with established inter-rater reliability. Fourth, we did not have data on the specific timing of VFSS relative to stroke onset (i.e., the number of days from onset to VFSS); this limits our ability to account for the potential influence of timing on swallowing outcomes, as dysphagia severity may evolve over the first days to weeks after stroke. Fifth, cognitive status was assessed using the MMSE alone, which does not dissect executive, visuospatial, or attentional domains that may be differentially relevant to each lesion. Sixth, termination bias should be acknowledged: because the VFSS protocol was terminated if patients exhibited significant difficulty, the semisolid data may underrepresent the most severely affected patients, particularly those who could not safely progress beyond the liquid trial. Seventh, this study was retrospective and single-institution, and the predominance of parietal lobe stroke in our cohort may reflect local referral patterns rather than the general epidemiology of post-stroke dysphagia. Prospective, multi-center validation in independent cohorts is warranted. Finally, we did not analyze pharyngeal-phase physiology in detail, and the contribution of pharyngeal pressure or laryngeal kinematics to the observed PAS differences remains to be explored [24].

Despite these limitations, our findings offer several clinically relevant observations. First, clinicians evaluating post-stroke dysphagia may benefit from considering lesion location when interpreting oral hesitation, since the same bedside sign appears to carry different weights as an aspiration warning. Second, chewing-focused assessment and rehabilitation may be particularly valuable in parietal stroke, where chewing function tracks with both oral hesitation and downstream airway compromise. Third, in parietal patients, the observation that oral hesitation was associated with an approximately three-fold increase in the odds of liquid aspiration may indicate a need for closer aspiration monitoring at the point of bedside detection, although confirmatory instrumental assessment remains essential. Fourth, in frontal stroke, the absence of detectable association between oral hesitation and aspiration risk in our limited sample may suggest a different clinical role for this sign, possibly related to cognitive or initiation factors, though this interpretation remains exploratory and requires confirmation in larger frontal cohorts.

## 5. Conclusions

Patterns of oral hesitation may differ between frontal and parietal lobe stroke. In parietal lobe stroke, oral hesitation was associated with chewing impairment and increased aspiration risk, and this association was robust to multivariable adjustment for age, sex, stroke type, and lesion side, suggesting a possible lesion-specific sensorimotor contribution. In frontal lobe stroke, we did not detect a significant association between oral hesitation and oral motor function or aspiration risk; however, the frontal cohort was significantly underpowered (*n* = 35), and these null findings should be considered exploratory rather than definitive. These preliminary observations suggest lesion-specific clinical implications of oral hesitation that may contribute to more personalized dysphagia assessment after stroke. Confirmation in larger, prospective, and multi-center cohorts is warranted.

## Figures and Tables

**Table 1:** Baseline characteristics of the study participants.

Variable	Frontal (*n* = 35)	Parietal (*n* = 207)	*p*-Value
Age, years (mean ± SD)	68.1 ± 15.0	71.7 ± 12.2	0.206
Female sex, *n* (%)	17 (48.6)	87 (42.0)	0.590
Lesion side, *n* (%)			<0.001 *
Left	17 (48.6)	111 (53.6)	
Right	12 (34.3)	94 (45.4)	
Bilateral	5 (14.3)	2 (1.0)	
Stroke type, *n* (%)			<0.001 *
Infarction	11 (31.4)	191 (92.3)	
Hemorrhage	24 (68.6)	16 (7.7)	
MMSE score (mean ± SD)	15.3 ± 10.5	15.6 ± 9.3	0.942
CDS total score (mean ± SD)	26.8 ± 19.6	22.4 ± 16.8	0.334

SD, standard deviation; MMSE, Mini-Mental State Examination; CDS, Clinical Dysphagia Scale. Continuous variables were compared with the Mann–Whitney U test. Categorical variables were compared with the chi-square test, or Fisher’s exact test when expected cell counts were <5. * *p* < 0.05.

**Table 2:** Clinical Dysphagia Scale subcomponent scores by lesion group.

CDS Subcomponent	Frontal (*n* = 35)	Parietal (*n* = 207)	*p*-Value
Lip sealing	2.21 ± 0.64	1.65 ± 0.59	<0.001
Chewing and mastication	2.15 ± 0.61	1.77 ± 0.61	0.001
Tongue protrusion	2.18 ± 0.63	1.77 ± 0.64	0.001
Laryngeal elevation	2.06 ± 0.49	1.80 ± 0.40	0.002
Reflex coughing	0.24 ± 0.43	0.21 ± 0.41	0.789
Residue in mouth	2.16 ± 0.45	1.75 ± 0.53	<0.001
Velar elevation	2.73 ± 0.45	1.31 ± 0.48	<0.001

Values are mean ± SD. *p*-values from Mann–Whitney U test.

**Table 3:** Correlation between chewing ability and swallowing outcomes, by lesion group, and MMSE-adjusted partial correlations in the frontal group.

Swallowing Outcome	Frontal ρ (*p*)	Parietal ρ (*p*)	Frontal MMSE-Adjusted ρ (*p*)
Oral hesitation, liquid	−0.023 (0.898)	0.185 (0.008) *	−0.011 (0.950)
Oral hesitation, semisolid	0.152 (0.389)	0.251 (<0.001) *	0.165 (0.351)
Vallecular residue	−0.118 (0.512)	0.301 (<0.001) *	−0.116 (0.519)
Pyriform residue	0.141 (0.435)	0.250 (<0.001) *	0.135 (0.453)

Values are Spearman’s ρ (*p*-value). MMSE-adjusted values are partial correlations controlling for MMSE score. * *p* < 0.05.

**Table 4:** Logistic regression of oral hesitation including a Group × MMSE interaction term.

Term	β	OR (95% CI)	*p*-Value
Oral hesitation, liquid			
Intercept	−0.934	0.39 (0.21 to 0.72)	0.003
Group (Frontal)	+4.806	122.2 (6.7 to 2243.9)	0.001
MMSE	−0.017	0.98 (0.95 to 1.02)	0.324
Group × MMSE	−0.118	0.89 (0.78 to 1.01)	0.068
Oral hesitation, semisolid			
Intercept	−0.948	0.39 (0.21 to 0.70)	0.001
Group (Frontal)	+2.603	13.5 (2.69 to 67.86)	0.001
MMSE	−0.004	1.00 (0.96 to 1.03)	0.795
Group × MMSE	−0.052	0.95 (0.87 to 1.03)	0.209

OR, odds ratio; CI, confidence interval. Firth’s penalized maximum likelihood estimation was used to address quasi-separation.

**Table 5:** Penetration-Aspiration Scale categories by lesion group.

Category	Frontal, Liquid	Parietal, Liquid	Frontal, Semisolid	Parietal, Semisolid
Normal (PAS = 1), *n* (%)	12 (36.4)	63 (31.7)	16 (69.6)	147 (73.1)
Penetration (PAS 2 to 5), *n* (%)	2 (6.1)	53 (26.6)	4 (17.4)	39 (19.4)
Aspiration (PAS 6 to 8), *n* (%)	19 (57.6)	83 (41.7)	3 (13.0)	15 (7.5)
Total, *n*	33	199	23	201

Values are *n* (%). PAS, Penetration-Aspiration Scale.

**Table 6:** Association between oral hesitation and airway invasion, by lesion group.

Outcome	Group	OH+ (%)	OH− (%)	OR (95% CI)	*p*-Value
Liquid aspiration (PAS 6–8)	Frontal	15/27 (55.6)	4/6 (66.7)	0.62 (0.10–4.01)	1.000
Liquid aspiration (PAS 6–8)	Parietal	27/42 (64.3)	56/157 (35.7)	3.25 (1.60–6.61)	0.001 *
Semisolid aspiration (PAS 6–8)	Frontal	3/18 (16.7)	0/5 (0.0)	2.48 (0.11–56.19) †	1.000
Semisolid aspiration (PAS 6–8)	Parietal	4/49 (8.2)	11/152 (7.2)	1.14 (0.35–3.75)	0.763
Semisolid any invasion (PAS ≥ 2)	Frontal	6/18 (33.3)	1/5 (20.0)	2.00 (0.18–22.06)	1.000
Semisolid any invasion (PAS ≥ 2)	Parietal	21/49 (42.9)	33/152 (21.7)	2.70 (1.36–5.36)	0.005 *

OH, oral hesitation; OR, odds ratio; CI, confidence interval; PAS, Penetration-Aspiration Scale. *p*-values from Fisher’s exact test; CIs were calculated using the Wald method on the log-OR scale. † Haldane–Anscombe correction (0.5) applied due to a zero cell. * *p* < 0.05.

## Data Availability

The data presented in this study are available on reasonable request from the corresponding author. The data are not publicly available due to privacy restrictions. Supplementary Tables S1 and S2 (sensitivity analysis within the frontal cohort) are provided as a separate file.

## Supplementary Materials

The following supporting information can be downloaded at: https://www.mdpi.com/article/10.3390/medicina62050918/s1, Table S1: Comparison of oral hesitation prevalence and airway invasion between hemorrhagic and ischemic subgroups within the frontal lobe stroke cohort; Table S2: Association between oral hesitation and airway invasion, stratified by stroke type within the frontal lobe stroke cohort.
