# Supplementary material for: Lesion-Specific Clinical Implications of Oral Hesitation After Stroke: A Comparative Study of Frontal Versus Parietal Lobe Lesions
Source: Medicina (Kaunas). 2026 May 9;62(5):918. doi: 10.3390/medicina62050918 (PMC13208578; doi:10.3390/medicina62050918)
Supplement: Supplementary file 1 [file medicina-62-00918-s001.zip › medicina-4293611-supplementary.pdf]

Table S1. Comparison of oral hesitation prevalence and airway invasion between hemorrhagic and ischemic subgroups within the frontal lobe stroke cohort.

Variable	Hemorrhage (n = 24)	Infarction (n = 11)	p-value
Oral hesitation, liquid	19/24 (79.2)	9/11 (81.8)	1.000
Oral hesitation, semisolid	15/24 (62.5)	9/11 (81.8)	0.435
Liquid aspiration (PAS 6–8)	11/23 (47.8)	8/10 (80.0)	0.131
Semisolid aspiration (PAS 6–8)	1/17 (5.9)	2/6 (33.3)	0.155

Values are n/N (%). PAS, Penetration-Aspiration Scale. p-values from Fisher's exact test.

Table S2. Association between oral hesitation and airway invasion, stratified by stroke type within the frontal lobe stroke cohort.

Outcome and subgroup	OH+, n/N (%)	OH−, n/N (%)	OR	p-value
Liquid aspiration (PAS 6–8)				
Hemorrhage (n = 23)	8/18 (44.4)	3/5 (60.0)	0.53	0.640
Infarction (n = 10)	7/9 (77.8)	1/1 (100.0)	—†	—†
Semisolid any invasion (PAS ≥ 2)				
Hemorrhage (n = 17)	4/12 (33.3)	1/5 (20.0)	2.00	1.000
Infarction (n = 6)	2/6 (33.3)	0/0 (—)	—†	—†

OH, oral hesitation; OR, odds ratio; PAS, Penetration-Aspiration Scale. p-values from Fisher's exact test. †Stable estimation not possible owing to a near-empty OH− subgroup (n = 1 for liquid aspiration; n = 0 for semisolid any invasion); descriptive percentages are shown.
